# Determinants of Dyslipidemia in Africa: A Systematic Review and Meta-Analysis

**DOI:** 10.3389/fcvm.2021.778891

**Published:** 2022-02-23

**Authors:** Mohammed S. Obsa, Getu Ataro, Nefsu Awoke, Bedru Jemal, Tamiru Tilahun, Nugusu Ayalew, Beshada Z. Woldegeorgis, Gedion A. Azeze, Yusuf Haji

**Affiliations:** ^1^College of Health Science and Medicine, Wolaita Soddo University, Wolaita Soddo, Ethiopia; ^2^College of Medicine and Health Science, Hawassa University, Hawassa, Ethiopia; ^3^College of Medicine and Health Science, Dilla University, Dilla, Ethiopia; ^4^Department of Anesthesia, Kotebe Metropolitan University, Addis Ababa, Ethiopia

**Keywords:** dyslipidemia, risk factors, lipid profile, Africa, abnormal lipid metabolism, metabolic syndrome, non-communicable disease, hypercholesterolemia

## Abstract

**Background:**

Dyslipidemia is a common public health problem in Africa. It has emerged as an important cardiovascular risk factor. It has been steadily increasing due to economic growth, urbanization, and unhealthy dietary pattern. Therefore, it is essential to identify determinants of dyslipidemia to prevent the condition and reduce its long-term sequel.

**Methods:**

Combinations of search terms with Boolean operators were used to retrieve studies from PubMed, EMBASE, Cochrane Database, Cinahl, Scopus, Mednar, and Google Scholar. The methodological quality of each article was evaluated based on the 2017 Joanna Briggs Institute (JBI) Critical Appraisal checklist for prevalence studies. After evaluation of each study against these criteria, studies with a minimum score of 7 or above out of 9 JBI checklists were included. We included articles presented in the English language. The Cochrane Q test was used to assess the heterogeneity across studies. The visual assessment of publication bias was done by creating a funnel plot. The possible causes of heterogeneity were explored by subgroup analyses. Egger's weighted regression test was used to assess the presence of publication bias. Statistical analyses were done by using the STATA software version 14.

**Result:**

A total of 24 articles involving 37,902 participants from 10 African countries were included. The overall pooled prevalence of dyslipidemia was 52.8 (95% *CI* 40.8–64.9). Individuals with a body mass index (BMI) >25.0 kg/m^2^ and waist circumference (WC) >94 cm were, respectively, 2.36 (95% *CI* (1.33–4.18), *p* < 0.001) and 2.33 (95% *CI* (0.75–0.29) *p* < 0.001) times more likely to develop dyslipidemia than those with lower values. Furthermore, patients with diabetes mellitus (DM) and hypertension (HTN) were 2.32 (95% *CI* (0.89–6.05) *p* < 0.001) and 2.05 (95% *CI* (1.31–3.21), *p* < 0.001) times more likely to present with dyslipidemia than non-diabetic patients and those without HTN.

**Conclusion:**

This study revealed that the prevalence of dyslipidemia is relatively high among study participants in African countries and the independent predictors of dyslipidemia were BMI >25.0 kg/m^2^, WC > 94 cm, raised blood glucose level, and raised blood pressure. Therefore, there should be a pressing public health measure to prevent, identify, and treat dyslipidemia with the special emphasis on obese, diabetic, and hypertensive patients.

## Background

Dyslipidemia is a state that occurs due to abnormalities in the plasma lipids. These include increased plasma total cholesterol (TC), increased low-density lipoprotein cholesterol (LDL-C), increased triglycerides (TGs), and reduced high-density lipoprotein cholesterol (HDL-C) levels. It presents either as an elevation of one of the lipids or high levels of a combination of the lipids (1, 2). The exact mechanism of dyslipidemia is not fully understood but is most likely multifactorial with genetic variations being shown to account for about 43–83% of the variability of plasma lipoprotein levels in a normal healthy population (3, 4). Dyslipidemia is an autosomal dominant disorder caused by mutations in the low-density lipoprotein receptor (*LDLR)* gene. As a result of defective cell-surface *LDLR*, clearance of LDL-C from plasma is reduced leading to the increased plasma levels of LDL-C and TC (5). Individuals with dyslipidemia have a 2-fold higher risk of developing cardiovascular diseases (CVDs) than those with normal lipid levels (6).

Both LDL-C and HDL-C regulate the amount of cholesterol in the body. An imbalance between the two can increase the risk of myocardial infarction and stroke (7). The increased levels of LDL-C aggravate the development of atherosclerosis, which is documented as the main risk factor for stroke, peripheral vascular, and ischemic heart disease (IHD) (8). However, HDL-C helps to remove cholesterol from the body, which decreases the risk of atherosclerotic CVD (6).

Though the burden of disease in Africa has been dominated by infectious diseases, countries in this region are undergoing a continuous demographic change leading to the increasing frequency of non-communicable diseases (NCDs) (9). Substantial changes in the health of the population marked by the rising burden of CVDs are set to overtake infectious diseases as the leading cause of death by 2030. This epidemiological shift is mainly driven by unhealthy lifestyles due to rapid urbanization and modernization. Recently, infectious agents are considered more frequently as causes of diseases that have been thought previously to be of non-infectious etiology, such as coronary heart disease. Additionally, lipopolysaccharide (LPS) affects the circulating macrophages and increases the production of free radicals. Free radicals are known to oxidize LDL-C and transform macrophages into foam cells, which are known to be crucial in the pathogenesis of atherosclerosis (10).

Dyslipidemia is responsible for more than half of the global IHD and more than 4 million deaths annually (11). It remains a common public health problem as abnormal serum lipid profile has been steadily increasing due to economic growth, urbanization, and unhealthy lifestyles (12). In Africa, the prevalence of dyslipidemia ranges from 5.2 (13) to 89.9% (14). The presentation of an abnormal lipid profile is common in individuals with central obesity, anti-retroviral therapy (ART), metabolic syndrome, insulin resistance, and type 2 diabetes mellitus (DM) (15). The duration of HIV treatment, advanced age, sex, low CD4 counts, smoking, alcohol consumption, depression (12, 15, 16), and a diet high in saturated fat, sedentary lifestyle, and obesogenic (17) are the major risk factors of dyslipidemia.

The prevalence of CVD is mainly result of risk factors, mostly hypertension (HTN), diabetes, and obesity (9). Dyslipidemia is also more common among patients with coexisting cardiovascular risk factors, such as HTN, diabetes, or HIV (10). A systematic review conducted in the Gambia on CVD risk factors revealed the prevalence of insufficient fruit and vegetable consumption, inadequate physical activity, and alcohol consumption was 77.8, 14.6, and 2.3%, respectively (18).

Most (80%) of the lipid disorders are associated with diet and lifestyle (17). The dietary habits of populations in low-to-middle-income countries (LMICs) have rapidly shifted to less-healthy diets (19). These consist of processed foods, away-from-home food intake, and increased use of edible oils and sugar-sweetened beverages (20).

In recent decades, the global pattern of unhealthy diets driving the occurrence of metabolic disorders and NCDs has become more important in LMICs because of the double burden of diseases in such countries that jointly constitute major causes of morbidity and mortality (21). In many African countries, surveillance and research on NCDs are still lacking (22). However, in the last decades, the number of NCDs-related deaths in the region have grown dramatically (9). This review will allow researchers to assess the disease burden of dyslipidemia in Africa where data are often scarce and populations are understudied. Assessing the prevalence of dyslipidemia can also aid researchers in predicting the future disease development of conditions contingent on dyslipidemia, such as CVD. Accurate data on dyslipidemia prevalence and risk factors can serve to inform health professionals, policy makers, and the public to manage and plan. However, different previous studies conducted on risk factors of dyslipidemia showed conflicting results. Understanding the context-specific factors associated with dyslipidemia and its potential implications are critical to designing effective interventions for the prevention and treatment of metabolic and cardiovascular effects of dyslipidemia.

## Methods

### The Study Protocol and Reporting

The Preferred Reporting Items for Systematic Reviews and Meta-Analyses (PRISMA) guidelines for literature search strategy, selection of studies, data extraction, and result reporting were followed while conducting this systematic review and meta-analysis. The eligibility criteria based on the Condition, Context, and Population (COCOPO) principle was adapted from the Joanna Briggs Institute (JBI) 2017 review guideline (23). Endnote (version X8) reference management software was used to download, organize, review, and cite related articles. The protocol of this review was not registered in the Prospero database.

#### Context

All eligible cross-sectional study, cohort study, comparative cross-sectional study, national survey, and a longitudinal study conducted on the prevalence and risk factors of dyslipidemia among patients with HIV, diabetes, HTN, and Lichen Planus, women on hormonal contraceptives, and general population in Africa were considered.

#### Condition

This review considered the studies that measured the outcome of interest-based prevalence and risk factors of dyslipidemia.

### Variables and Measures

The primary outcome of this study was dyslipidemia. The case definitions of dyslipidemia were increased TC ≥5.17 mmol/L (≥200 mg/dl); high LDL-C ≥3.36 mmol/L (≥130 mg/dl), increased TG ≥1.7 mmol/L (≥150 mg/dl); and low HDL-C <1.03 mmol/L (<40 mg/dl) for men, <1.3 mmol/L (<50 mg/dl) for women. Study subjects who met one or more of the above criteria were categorized as having dyslipidemia. Participants who were on a lipid-lowering agent were also classified as having dyslipidemia (24, 25). Identified determinants of dyslipidemia were defined as follows: abdominal obesity was defined as WC >94 cm for men and >88 cm for women (26). BMI was classified as underweight (<18.5 kg/m^2^), normal (18.5–24.9 kg/m^2^), overweight (25.0–29.9 kg/m^2^), or obese (≥30 kg/m^2^) (27). Participants were considered as hypertensive if blood pressure (BP) is ≥140/90 mmHg. In this study, high fasting blood glucose (FBG) was defined as ≥5.6 mmol/L (≥100 mg/dl) or if the patient is on treatment for diabetes (28).

### Inclusion Criteria

We included all studies that fulfilled the above case definition of dyslipidemia. However, we considered studies that were conducted on determinants of metabolic syndrome and hypercholesterolemia if risk factors of dyslipidemia were identified. In such a case, we carefully checked its compliance with the case definition before considering it for the final analysis. Furthermore, full text and/or open access articles, a study conducted in Africa, published and unpublished studies are written in the English language before August 8, 2021, were included.

### Study Design and Search Strategy

The search was limited to studies published in the English language from January 2000 to July 20, 2021. For published studies, the initial restricted search of PubMed, EMBASE, Cochrane Database, Cinahl, Scopus, Mednar, and Google Scholar were undertaken. Thereafter, unpublished studies were searched from Addis Ababa University and Jimma University institutional repositories. Then, a second search term using all identified keywords and index terms was undertaken. Third, the reference list of all identified reports and articles was searched for extra studies. We searched the database with terms including:

“Dyslipid^*^,” “Dyslipidemia,” ”Dyslipidaemia,” “metabolic syndrome,” “hypercholesterol^*^,” “hypercholesterolemia,” hypercholesterolemia “prevalence,” “associated factors,” “general population,” “Sub-Saharan Africa,” and “Africa” for the articles published in English before August 8, 2021. The search detail of this systematic review with meta-analysis was uploaded as dataset 1.

### Data Quality Control Measures

The methodological quality of each article was evaluated based on the 2017 JBI Critical Appraisal checklist for prevalence studies (23). After the evaluation of each study against these criteria, studies scoring 7 or above were included. On the other hand, studies scoring below 7 out of 9 criteria of Critical Appraisal instruments for prevalence studies were excluded. The quality of each study was evaluated independently by two authors (MS. O. and G. A.). The discrepancy was solved by the discussion with the third independent reviewer (BZ. W.).

### Data Extraction

The data were extracted by two independent authors (MS. O. and Y. H.) using a standard and piloted form. The extracted data were the last name of the author, year of publication, country, region, study participant, frequency or prevalence of the disease, LDL-C data, and risk factors dataset 2. Discrepancies were discussed and solved by consensus or by a third reviewer (G. A.).

### Statistical Analysis

All statistical data analyses were done by using Stata version 14.0 (Statacorp. LP, College Station, TX, USA). A random effects meta-analysis based on the DerSimonian and Laird approach was used to pool the prevalence of dyslipidemia in Africa. The presence of statistical heterogeneity was checked using Higgins I-squared (*I*^2^) statistics and Cochran's-Q test. Accordingly, heterogeneity was classified as low, moderate, or high when the values of *I*^2^ were 25, 50, and 75%, respectively (13). The potential sources of heterogeneity were explored by meta regression and subgroup analysis. Publication bias was viewed graphically by the funnel plots and tested through Egger© and Begg© tests (29). Sensitivity analysis was done to identify the effect of a single study on the overall estimate.

## Result

### Search Results

**Figure 1:**
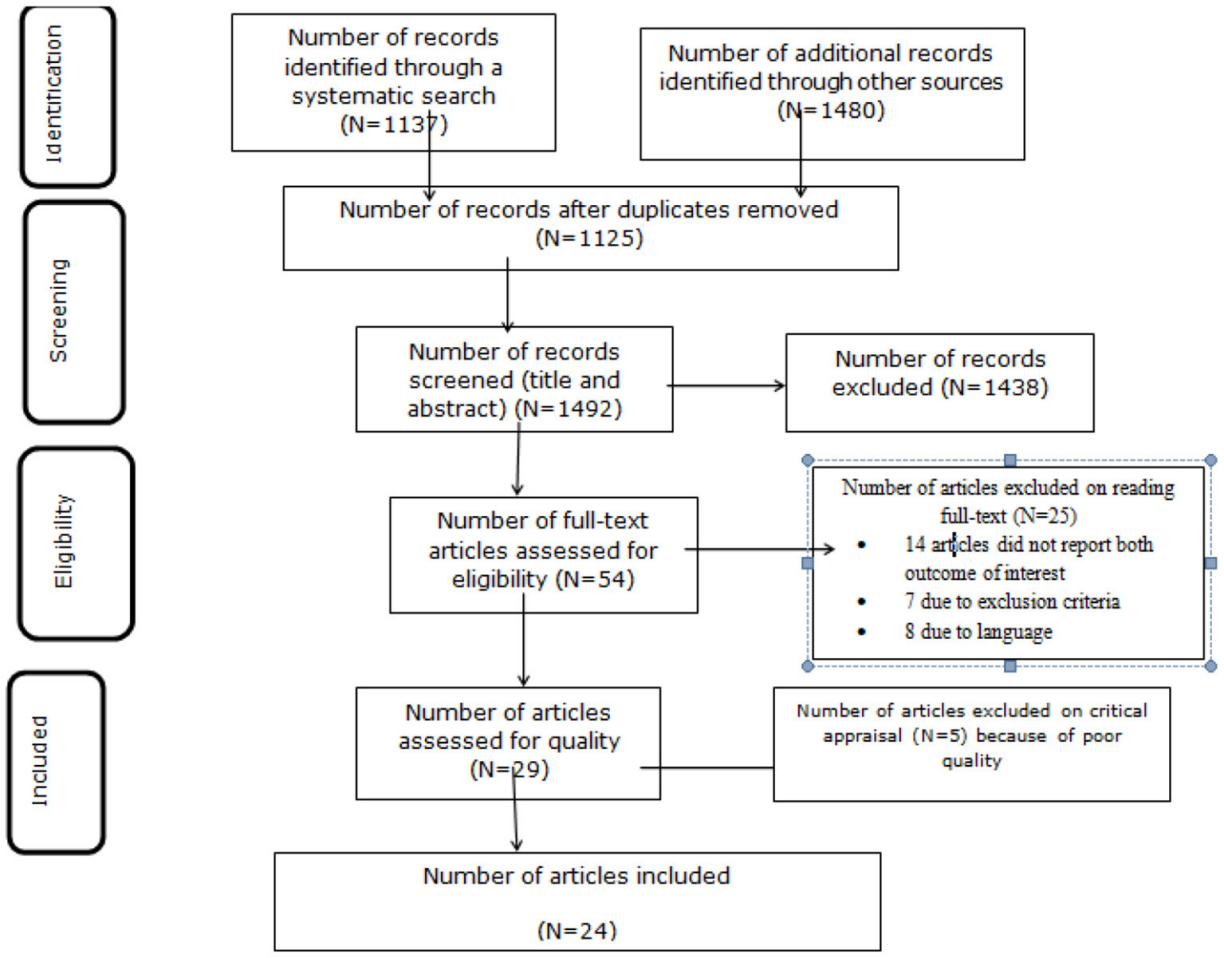
PRISMA flow diagram of dyslipidemia in Africa, 2021.

Initially, a total of 1,137 studies were retrieved from the electronic databases and 1,480 articles were retrieved through manual searching. The total number of articles retrieved through an electronic database and manual searching was 2,617. From this, 1,125 were duplicates and removed from this systemic review. The remaining 1,492 articles were screened by their title and abstract and 1,438 irrelevant studies were removed. Then, 54 full-text articles were assessed for eligibility and 14 of them were excluded on the account of not reporting the outcome of interest, 7 studies were excluded due to exclusion criteria, and 8 studies were excluded due to language. Thereafter, 5 articles were excluded upon evaluation against the critical appraisal checklist of prevalence studies (23). Finally, 24 studies were included in this systematic review with meta-analysis (Figure 1).

### Study Characteristics

A total of 24 articles from 10 African countries were included in this systematic review and meta-analysis. Out of 37,902 study subjects, 12,035 patients had dyslipidemia. The sample size across the studies ranges from 90 (30) to 10,690 (31). Out of 24 articles, nine were conducted in Ethiopia, four in South Africa, three in Nigeria, and two others in Kenya. The remainders of the studies of interest were conducted in Mozambique, Zambia, Senegal, Uganda, Togo, and Malawi. The highest prevalence of dyslipidemia was reported in a study from South Africa (89.9%) (14), and from Kenya (85.6%) (32). On the other hand, the lowest prevalence of dyslipidemia of 5.2% was reported in a study from Ethiopia (13). The highest percentage of LDL-C data was reported in a study from Ethiopia (79.7%) (33) (Table 1).

**Table 1:** Prevalence of dyslipidemia based on health status and for the general population in Africa, 2021.

**Authors**	**Year**	**Country**	**Region**	**Study participant**	**Study design**	**Sample size**	**Frequency**	**LDL-C in %**
Kemal et al. (16)	2020	Ethiopia	East Africa	Patients on ART	Cross-sectional	353	264	31.2
Kiplagat et al. (32)	2017	Kenya	East Africa	Type-2 DM	Cross-sectional	265	208	Not reported
Abdu et al. (33)	2020	Ethiopia	East Africa	Patients with *H. Pylori*	Cross-sectional	369	286	79.7
Haile et al. (34)	2020	Ethiopia	East Africa	Diabetes patients	Cross-sectional	248	169	28.6
Bekele et al. (35)	2017	Ethiopia	East Africa	Diabetes patients	Cross-sectional	224	150	43.8
Sufa et al. (8)	2019	Ethiopia	East Africa	General population	Cross-sectional	365	127	34.8
Okpala et al. (30)	2019	Nigeria	West Africa	Lichen Planus patients	Cross-sectional	90	15	Not reported
Yusuf et al. (36)	2015	Nigeria	West Africa	Lichen Planus patients	Cross-sectional	180	51	Not reported
Fikremariam et al. (37)	2018	Ethiopia	East Africa	Diabetes patients	Cross-sectional	112	94	Not reported
Ditorguéna et al. (38)	2019	Togo	West Africa	General population	Cross-sectional	746	450	0.7
Amberbir et al. (39)	2018	Malawi	Central Africa	HIV patients	Cross-sectional	554	86	Not reported
Tilahun et al. (2)	2021	Kenya	East Africa	HIV patients	Cross-sectional	564	265	26.6
Ciccacci et al. (40)	2021	Mozambique	Southern Africa	DM and HTN patients	Cross-sectional	885	410	Not reported
Hamooya et al. (41)	2021	Zambia	Central Africa	HIV patients	Cross-sectional	1,108	293	Not reported
Gebreegziabiher et al. (17)	2021	Ethiopia	East Africa	General population	Cross-sectional	370	204	49.5
Doupa et al. (42)	2014	Senegal	West Africa	General population	Cross-sectional	1,329	880	66.2
Asiki et al. (43)	2015	Uganda	East Africa	General population	Cross-sectional	7,809	5,567	5.2
Tadewos et al. (44)	2012	Ethiopia	East Africa	HIV	Comparative	113	67	48.7
Ayoadea et al. (45)	2020	Nigeria	West Africa	HTN patients	Cross-sectional	544	326	27.9
Dave et al. (14)	2016	South Africa	Southern Africa	HIV	Comparative	957	861	55.1
Jamieson et al. (31)	2017	South Africa	Southern Africa	HIV	Cohort study	10,690	595	Not reported
Pitso et al. (46)	2021	South Africa	Southern Africa	Diabetes	Cross-sectional	143	120	20.9
Innes et al. (47)	2016	South Africa	Southern Africa	HIV	Longitudinal	96	38	26
Gebreyes et al. (13)	2018	Ethiopia	East Africa	General population	National survey	9,788	509	14.1

^*^ART, anti-retroviral therapy and DM, diabetes mellitus.

### Prevalence of Dyslipidemia

**Figure 2:**
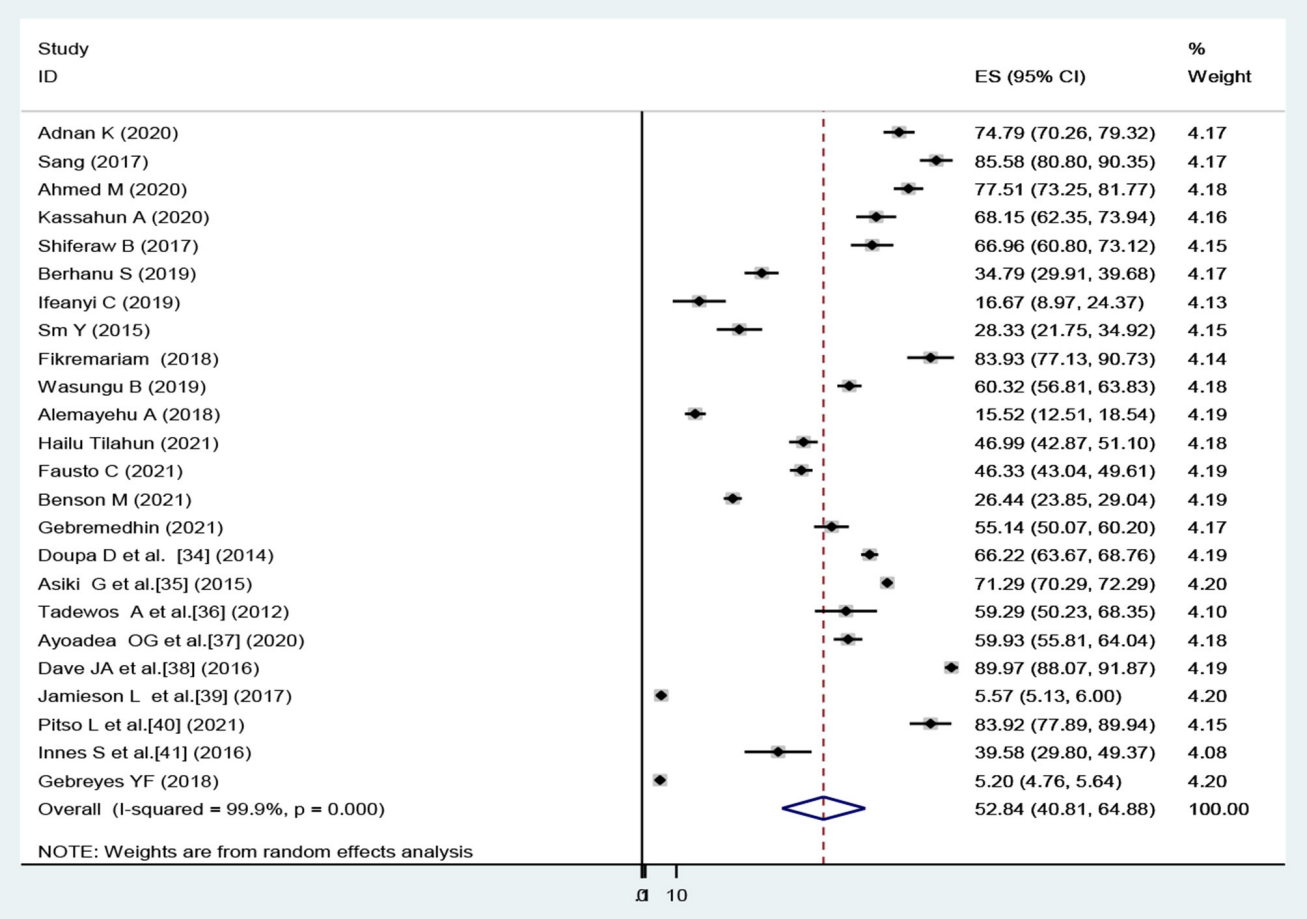
Overall pooled prevalence of dyslipidemia by African regions, 2021.

The overall pooled prevalence of dyslipidemia in Africa using the fixed effect model was 16.5 (95% *CI* (16.0–16.5). When using the fixed effect model, the pooled effect size of dyslipidemia showed a significant heterogeneity (*I*^2^) of 99.9% (*p* < 0.001). As a result, we determined the final pooled prevalence with a random-effect model to control the observed unevenness. The final pooled prevalence of dyslipidemia was 52.8 (95% *CI* (40.8–64.9) (Figure 2).

### Heterogeneity

The subgroup analysis by the category of population and regions was done to handle the heterogeneity. In addition, we performed meta regression with sample size, prevalence, and year of publication. The details of subgroup analysis and meta regression were mentioned below.

### Subgroup Analysis by the Category of Population or Study Participant

Subgroup analyses based on the underlying diseases and the countries revealed a marked variation in the prevalence of dyslipidemia. When we look at variation based on the category of study participant or population, the prevalence of dyslipidemia was the highest among the category of studies conducted on a group of participants with NCDs (patients with DM, HTN, and DM and HTN) [70.6 (95% *CI* (58.0–83.2)] and the general population [48.8 (95% *CI* (13.7–84.0)]. On the other hand, the lowest prevalence of dyslipidemia {43.7 [95% *CI* (18.9–68.5)]} was reported among studies conducted on a group of participants with infectious diseases (patients with HIV, *H. Pylori*, and Lichen Planus) (Table 2).

**Table 2:** The pooled prevalence of dyslipidemia, 95% *CI*, and heterogeneity estimate with a *p*-value for subgroup analysis.

**Variable**	**Characteristics**	**Pooled prevalence 95% (CI)**	***I*^2^ (%)**
Category of population	Patients with NCDs	70.6 (58.0–83.2)	97.8
	Patients with infectious disease	43.7 (18.9–68.5)	99.9
	General population	48.8 (13.7–84.0)	100

### A Subgroup of Analysis by the Region

**Figure 3:**
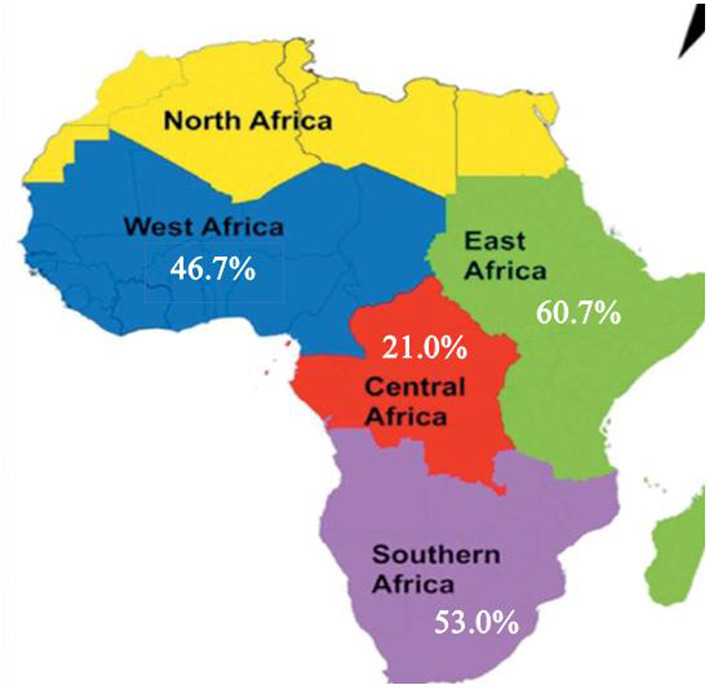
Subgroup analyses on the pooled prevalence of dyslipidemia by African regions, 2021.

A subgroup analysis by the region showed that there was marked variation across the regions of the African continent. The highest prevalence of dyslipidemia found in East Africa was 60.8 [(95% *CI*: 35.5–86.0), *I*^2^ = 99.9, *p* < 0.001] and Southern Africa was 53.1 (95% *CI*: 6.1–99.4), *I*^2^ = 100%, *p* < 0.001). The pooled prevalence of dyslipidemia in western Africa and Central Africa was 46.7 (95% *CI*: 32.4–60.9), *I*^2^ = 98.3, *p* < 0.001) and 21.0 (95% *CI*: 10.3–31.7), *I*^2^ = 96.5, *p* < 0.001), respectively. Out of 24 included studies, 12 studies were from East Africa, 5 studies were from western Africa, 5 studies were from southern Africa, and two studies were from central Africa. But, not a single study was included from the northern African region (Figure 3).

### Meta Regression

We applied meta regression to identify the source of heterogeneity. Accordingly, random-effects meta regression was conducted by considering the year of publication, prevalence, and sample size as covariates. The analysis indicated that heterogeneity was explained by prevalence (*p* = 0.02) (Table 3).

**Table 3:** Meta regression analysis of factors affecting between study heterogeneity.

**Heterogeneity source**	**Coef**.	**Std. Err**.	**T**	**P>t**	**[95% Conf. Interval]**	
Year	0.01	0.13	0.10	0.92	−29.02	0.26
Prevalence	0.02	0.01	2.60	0.02^*^	−0.01	0.05
Sample size	0.00	0.00	0.90	0.38	−0.01	0.01

* *Statistically significant variables at P value < 0.05*.

### Sensitivity Analysis

A sensitivity analysis was executed by removing studies step-by-step to evaluate the effect of a single study on the whole estimate. As shown in Table 4, no study exhibited an important effect on the final pooled prevalence of dyslipidemia.

**Table 4:** Sensitivity analysis of dyslipidemia among included study in Africa, 2021.

**Study omitted**	**Country**	**Study participant**	**Estimate**	**[95% CI]**	
Kemal et al. (16)	Ethiopia	Patients on ART	51.9	39.7	64.1
Kiplagat et al. (32)	Kenya	Type-2 DM	51.4	39.3	63.6
Abdu et al. (33)	Ethiopia	Patients with *H. Pylori*	51.8	39.6	63.9
Haile et al. (34)	Ethiopia	Diabetes patients	52.2	39.9	64.4
Bekele et al. (35)	Ethiopia	Diabetes patients	52.2	40.0	64.5
Sufa et al. (8)	Ethiopia	General population	53.6	41.3	65.9
Okpala et al. (30)	Nigeria	Lichen Planus patients	54.4	42.1	66.7
Yusuf et al. (36)	Nigeria	Lichen Planus patients	53.9	41.6	66.2
Fikremariam et al. (37)	Ethiopia	Diabetes patients	51.5	39.3	63.7
Ditorguéna et al. (38)	Togo	General population	52.5	40.3	64.8
Amberbir et al. (39)	Malawi	HIV patients	54.5	42.0	66.9
Tilahun et al. (2)	Kenya	HIV patients	53.1	40.8	65.4
Ciccacci et al. (40)	Mozambique	DM and HTN patients	53.1	40.8	65.4
Hamooya et al. (41)	Zambia	HIV patients	54.0	41.6	66.4
Gebreegziabiher et al. (17)	Ethiopia	General population	52.7	40.5	65.0
Doupa et al. (42)	Senegal	General population	52.3	40.1	64.4
Asiki et al. (43)	Uganda	General population	52.0	41.9	62.1
Tadewos et al. (44)	Ethiopia	HIV	52.6	40.3	64.8
Ayoadea et al. (45)	Nigeria	HTN patients	52.5	40.3	64.8
Dave et al. (14)	South Africa	HIV	51.2	40.0	62.5
Jamieson et al. (31)	South Africa	HIV	54.9	40.0	71.9
Pitso et al. (46)	South Africa	Diabetes	51.5	39.3	63.7
Innes et al. (47)	South Africa	HIV	53.4	41.1	65.7
Gebreyes et al. (13)	Ethiopia	General population	54.9	38.0	71.8
Combined			52.8	40.8	64.9

### Publication Bias

**Figure 4:**
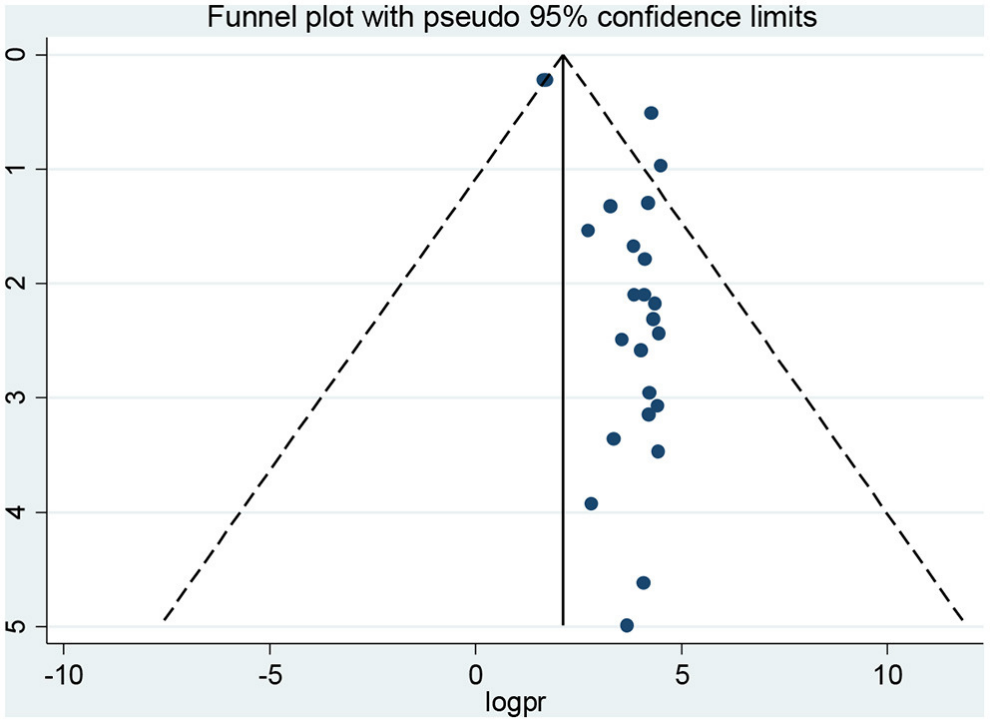
Funnel plots for publication bias for the prevalence of dyslipidemia in Africa, 2021.

**Figure 5:**
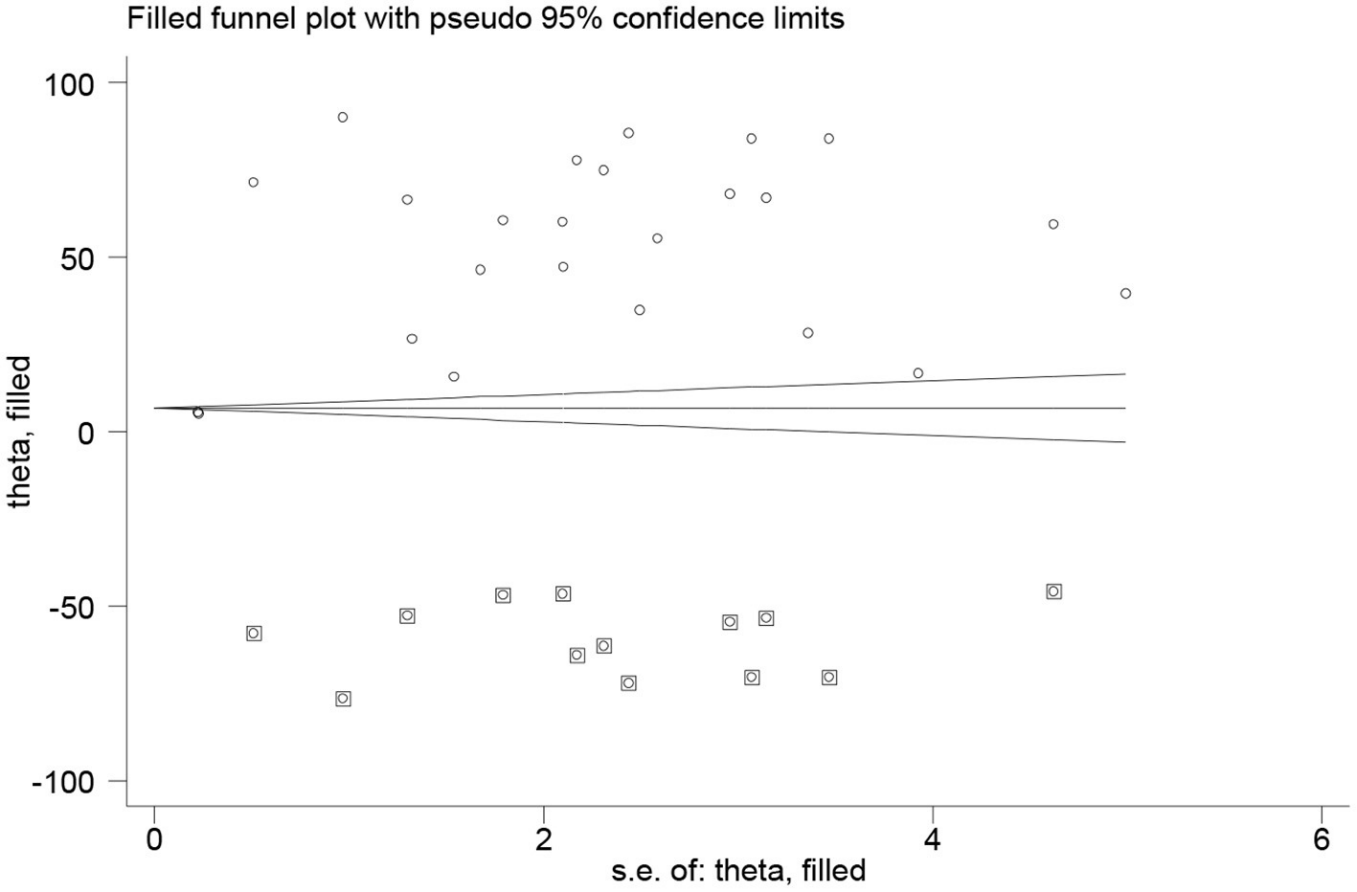
Trim and fill analysis for the prevalence of dyslipidemia in Africa, 2021.

As indicated in Figure 4, the visual inspection of the funnel plot showed an asymmetrical distribution. When objectively evaluated against the Egger© regression test and an adjusted Begg© rank correlation test at a 5% significance level, we found *p* < 0.001 and 0.009, respectively. This confirmed that there was evidence of publication bias in this systematic review with meta-analysis. Therefore, Duval and Tweedie's trim and fill analysis (metatrim) using the random-effects model to see the impact of publication bias through the assumption that the funnel plot asymmetry was solely caused by publication bias might not hold for this dataset. The trim and fill analysis showed the presence of 13 unpublished studies. Considering these studies in calculating the pooled prevalence yields, an estimated pooled prevalence of dyslipidemia, which is adjusted for publication bias was found to be 13.38% [95% *CI* (0.52–26 24); *p* = 0.04] (Figure 5).

**Figure 6:**
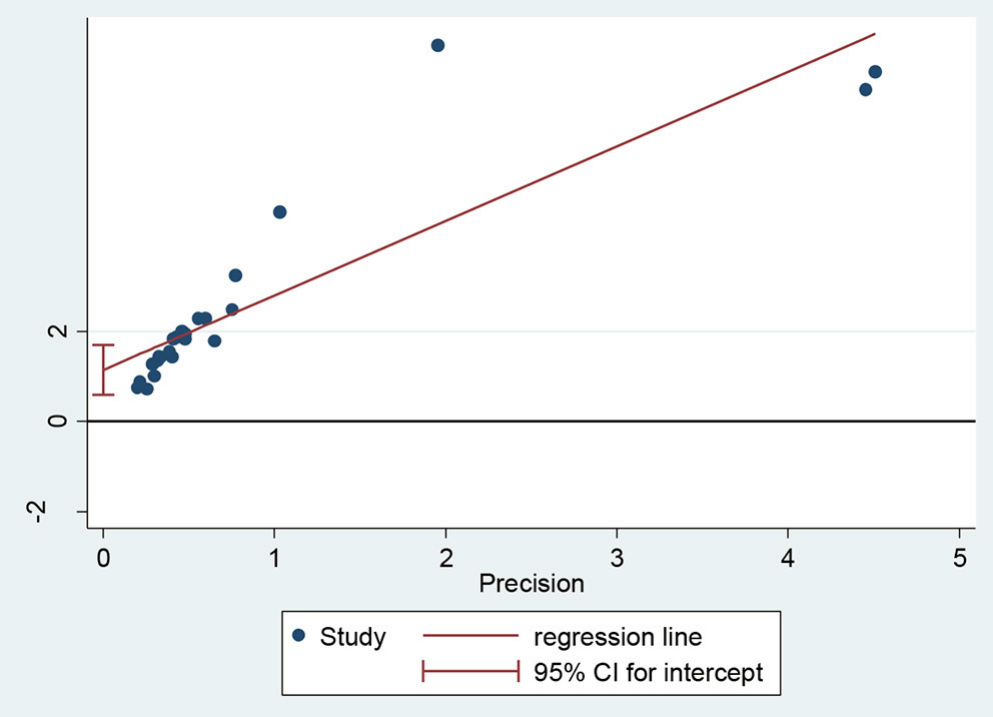
Regression graph of dyslipidemia in Africa, 2021.

As shown in the regression graph (Figure 6), the estimated bias coefficient (intercept) is 1.14 with an SE of 0.26, giving a *p* < 0.001 and *CI* of 0.58–1.70. Thus, the test provides strong evidence for small-study effects.

**Figure 7:**
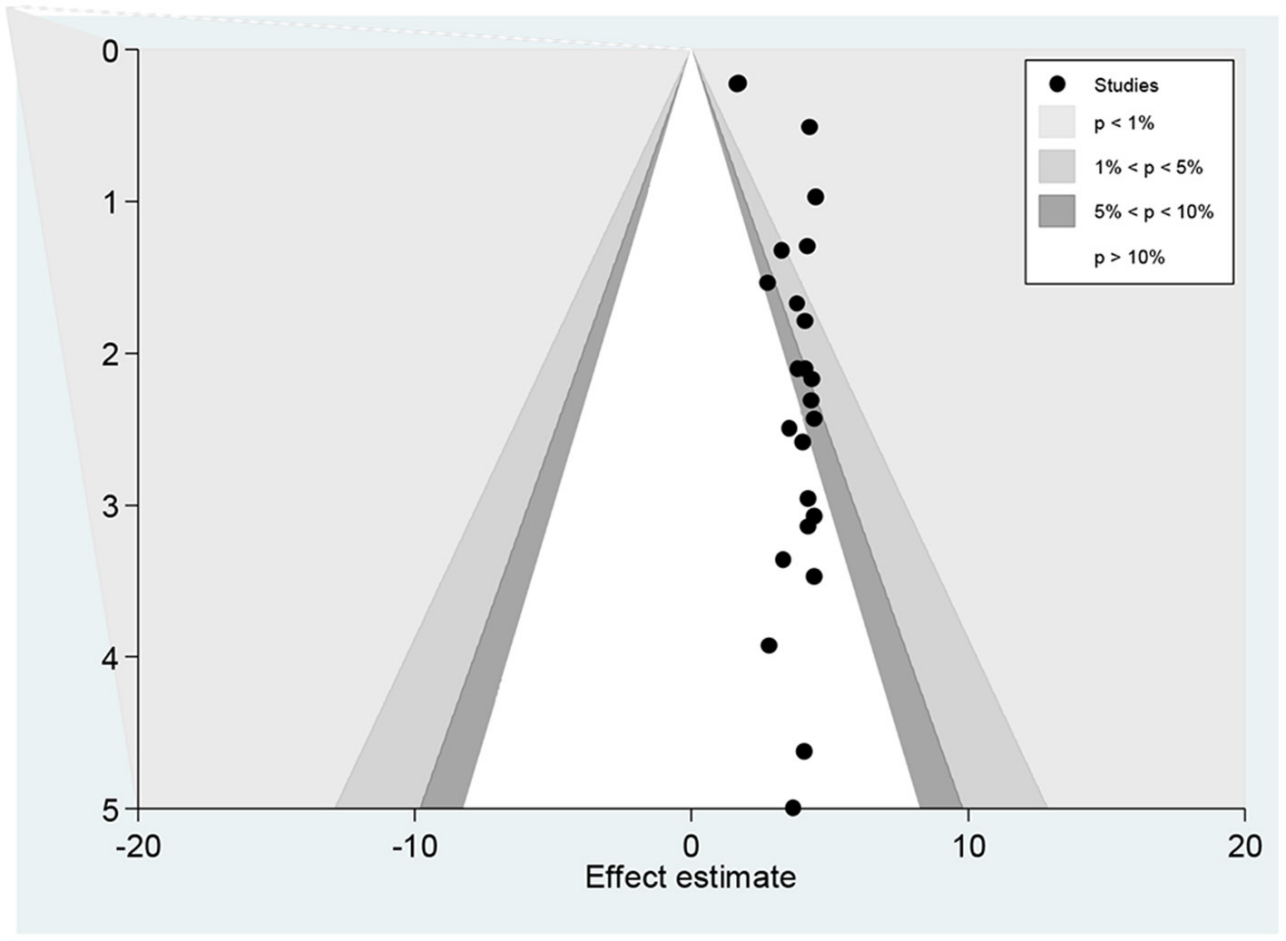
Counter enhanced funnel plots for publication bias for the prevalence of dyslipidemia in Africa, 2021.

**Figure 8:**
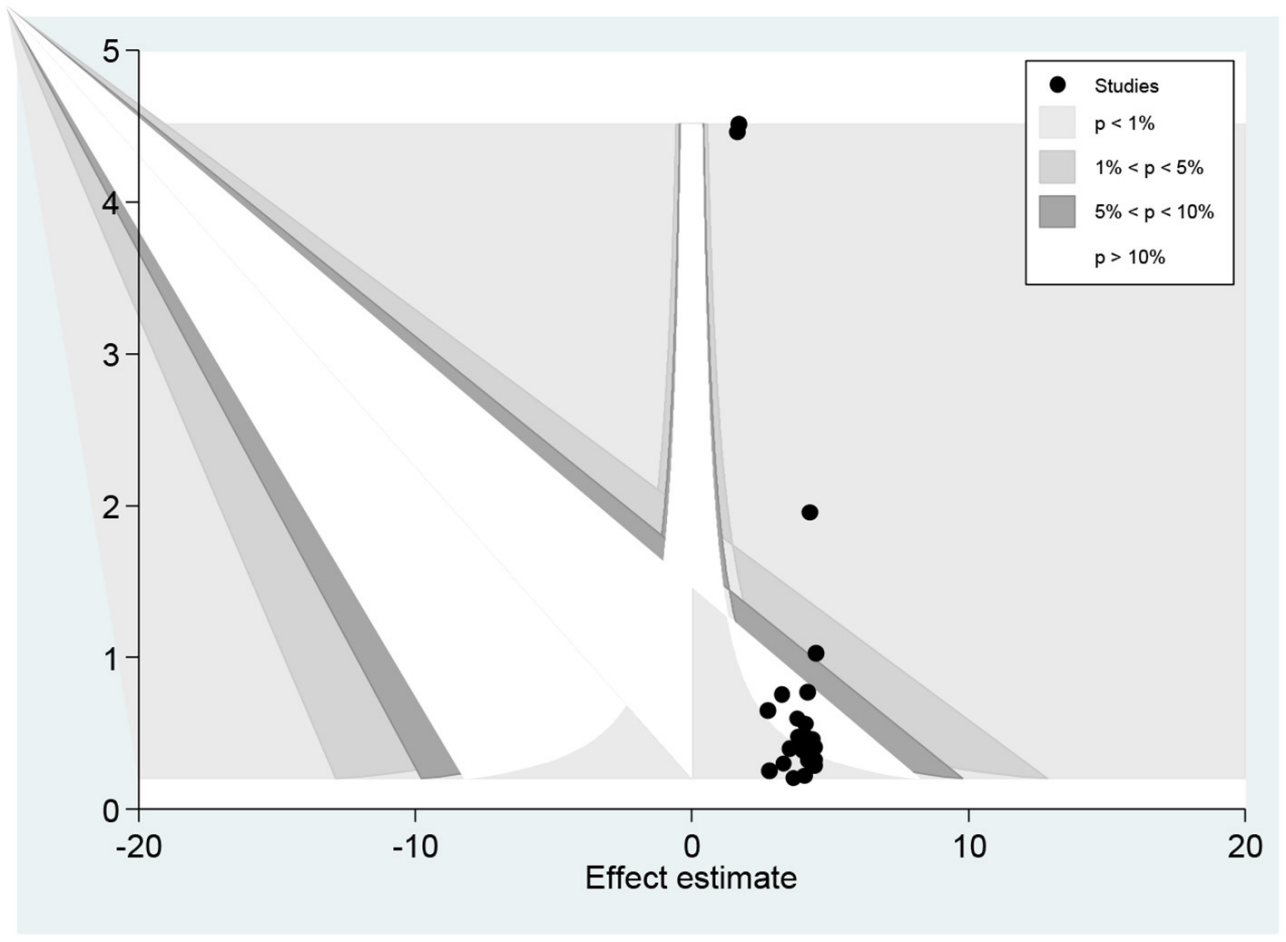
Meric inverse counter enhanced funnel plots of publication bias for the prevalence of dyslipidemia in Africa, 2021.

The funnel plot showed a small study effect; Hhowever, the counter-enhanced funnel plot showed that small studies were found both in the area of statistical significance (shaded area) and non-statistical significance (white area). So, the asymmetry may have been caused by a number of other factors rather than the publication bias (Figure 7). Similar findings also occurred when we performed the metric counter-enhanced funnel plot (Figure 8).

### Determinants of Dyslipidemia in Africa

Furthermore, 11 variables were extracted to identify factors associated with dyslipidemia. Of these variables, 9 of them (sex, medication adherence, educational status, FBS, BP, BMI, WC, marital status, and sedentary life) were significantly associated with dyslipidemia at *p* < 0.05.

Patients with BMI >25 kg/m^2^ and patients with WC >94 cm were about 2.36 and 2.3 times more likely to develop dyslipidemia, as compared with their counterparts (odds ratio (*OR*): 2.36 (95% *CI* (1.33–4.18), *p* < 0.001, *I*^2^: 89.6%) and *OR*: 2.33 [95% *CI* (0.75–7.29), *p* < 0.001, *I*^2^: 89.6%)]. Patients with DM were about 2.3 times more likely to have dyslipidemia, as compared with non-diabetic patients {*OR*: 2.32 [95% *CI* (0.89–6.04)] *p* < 0.001, *I*^2^: 88.1%}. Furthermore, known smoker patients were about 132% more likely to develop dyslipidemia, as compared with non-smoker patients {*OR*: 1.32 [95% *CI* (0.74–2.35)] *p* = 0.05, *I*^2^: 80.7%].

This study also showed that patients with HTN were about 2 times more likely to have dyslipidemia when compared with non-hypertensive patients {*OR*: 2.05 [95% *CI* (1.31–3.21)], *p* < 0.001, *I*^2^: 85%}. Furthermore, patients who had no history of alcohol consumption and sedentary lifestyles were about 20% less likely to develop dyslipidemia than their counterparts {*OR*: 0.862 [95% *CI* (0.68–1.11)] and *OR*: 0.80 [95% *CI* (0.63–1.10)]}, respectively (Table 5).

**Table 5:** Factors associated with dyslipidemia in Africa, 2021.

**Determinants**	**Comparison**	**Number OF studies**	**Sample size**	**OR (95% CI)**	***P*-value**	***I*^2^ (%)**	**Heterogeneity test (*P*-value)**	**Egger©test (*P*-value)**
Sex (16, 32–35, 39–41)	Male vs. female	8	3,596	0.86 (0.67–1.08)	<0.001^*^	72	0.19	0.17
Smoking (16, 32, 34)	Yes vs. no	3	809	1.32 (0.74–2.35)	0.47	0.0	0.35	0.18
Medication adherence (16, 32)	No vs. yes	2	561	0.80 (0.52–1.23)	0.03^*^	79.9	0.31	^-^
Educational status (8, 32, 34, 35)	Illiterate vs. literate	4	1,044	1.19 (0.90–1.57)	0.01^*^	75	0.22	0.95
FBS (17, 32, 34, 40)	Positive vs. negative	4	1,622	2.32 (0.89–6.05)	<0.001^*^	88.1	0.09	0.10
Residence (33–35, 39)	Rural vs. urban	4	1,395	0.75 (0.40–1.40)	0.14	49.8	0.37	<0.001^*^
BP (16, 17, 32–35, 40)	High vs. low	7	2,507	2.05 (1.31–3.21)	<0.001^*^	75.4	<0.001	0.09
Alcohol (16, 32, 34, 38)	Yes vs. no	5	1,924	0.86 (0.68–1.09)	0.49	41.3	0.208	0.20
BMI (16, 17, 32, 34, 35, 37, 38, 40)	Yes vs. no	8	3,436	2.36 (1.33–4.18)	<0.001^*^	89.6	<0.001	0.58
WC (17, 33, 40)	≥94 vs. <94 cm	3	393	2.33 (0.75–7.29)	<0.001^*^	93.6	0.15	0.28
Marital status (8, 32, 34)	Married vs. single	3	841	1.174 (0.65–2.10)	<0.001	75	0.59	0.95
Sedentary life (16, 32, 34, 38)	Yes vs. no	4	1,555	0.80 (0.63–1.03)	0.04^*^	64.9	0.08	0.41

*FBS, fasting blood sugar; BP, blood pressure; BMI, body mass index; OR, odds ratio; vs., versus.*

- *Insufficient observation. Egger test P-value was not calculated if degree of freedom is zero due to insufficient number of study.*

* *Statistically significant variables at P-value < 0.05*.

## Discussion

Dyslipidemia has emerged as an important cardiovascular risk factor in African countries (17). It is characterized by LDL-C increment, TG elevation, and HDL-C decrement or combined. The *LDLR* positioned on chromosome 19p13.2 plays a significant role in lipoprotein metabolism by mediating the uptake of cholesterol through the binding and subsequent cellular uptake of apolipoprotein-E and B- constituting lipoproteins. Mutations have been detected in different domains of the *LDLR* which have a distinct effect on LDLR structure and function. ATP-binding cassette A1 (*ABCA1*) plays a critical role in the reverse cholesterol transport system. Mutation in *ABCA1*, that encodes this protein, along with genes responsible for their transcription regulation, can lead to abnormality in the metabolism of lipids (48). Apolipoprotein A5 (*APOA5*) has been shown to be a key regulator of plasma TGs and there are several single nucleotide polymorphisms (SNPs) associated with the *APOA5* gene (49). Future research should address the lack of LDL-C oxidation data generated by existing studies.

The prevalence of dyslipidemia in this review is higher than the finding of the study conducted among the hormonal contraceptive users from April to June 2014 at three health centers and one hospital in Harar town, Eastern Ethiopia (34.8%) (8). However, the present finding is lower than the findings of the study in Jimma, Southwestern Ethiopia (68.1%) (34), and South Africa (67.3%) (50). The finding of this review was also lower than the finding of a study done in Kembata, Southern Ethiopia (65.5%) (35), and Mekelle city, Northern Ethiopia (66.7%) (17). The higher prevalence of dyslipidemia might be attributed to the rapid urbanization, improved socioeconomic status, a change in the power of work, inadequate physical activity, and alteration in dietary habits.

The present study showed that the raised FBG was positively associated with dyslipidemia, which agrees with many previous studies (8, 51, 52). This might be because DM can cause a range of derangements in oxidation or reduction in lipid metabolism, which could in turn be responsible for the accumulation of lipid particles (34). We also revealed that the raised BP level was an independent predictor of dyslipidemia, which is consistent with the study conducted in Germany (52); but, the findings of this review disagree with the findings of the study reported in Iraq (53). This variation might be due to differences in the underlying disease, socio-demographic factors, genetic predisposition, dietary factors, and inadequate physical activity, which results in a higher incidence of metabolic abnormalities.

In the current review, BMI was found to have a positive association with dyslipidemia, which is consistent with many previous studies (16, 51, 54, 55). This finding is also similar to studies conducted in Ethiopia (44), Uganda (56), and Tanzania (57). However, previous works have doubted the reliability of BMI as an indicator of obesity (58, 59). Moreover, in young healthy adults, BMI and other surrogate indices of fatness, such as waist-to-height ratio and body adiposity index provide a poor prognosis of fat mass (60, 61). The observed association in other studies can be supported by the occurrence of ART associated lipodystrophy, which may be accompanied by the loss of peripheral subcutaneous fat (62). The other differences may be because the current review was conducted on participants with underlying different types of disease while other studies conducted only on patients with HIV and DM.

In our review, abdominal obesity or WC ≥ 94 cm was significantly associated with dyslipidemia, which agrees with other studies (34, 63). So far, anthropometric measurements, such as BMI, have been the most widely used instrument to measure general obesity. However, BMI does not account for the variation in body fat distribution and abdominal fat (64, 65). Excess of intra-abdominal fat causes a greater risk of morbidity than the overall fat accumulation (66). Thus, WC has been by far the best indicator of both intra-abdominal and total fat (67). Generally, altered anthropometric (68) and adipose tissue size have been associated with dyslipidemia in persons with and without HIV (69, 70). We found that the prevalence of dyslipidemia was almost equally determined by both BMI and WC. This might be due to a high chance of increasing concentrations of different lipid particles with increased BMI. Therefore, the combination of different anthropometric measurements should be used to evaluate the health condition of the patient or population.

Similar to the current review, studies conducted in South Africa (71), Cameroon (72) showed that being the female gender was not associated with a higher probability of presenting with dyslipidemia. In contrast to our review, the findings of a study done in Tanzania showed that being the female gender was an independent predictor of dyslipidemia (57). The discrepancy is more likely due to gender-related exercise, habits, and biological differences between men and women (27). Moreover, this study also found that lower odds of dyslipidemia were found among participants who have attained a lower level of education when compared with those who attended college and above. This is in agreement with other studies (41, 73). In contrast, other studies found contradicting results (74, 75). This may be due to differences in lifestyle.

In this study, sex, increased BMI, increased BP, and sedentary lifestyles were significantly associated with the increased risk of dyslipidemia. As all are modifiable risk factors except sex, it is essential to deliver behavioral interventions on the identified risk factors. Dietary modification is one of the most important lifestyle changes that have been shown to significantly decrease the risk of CVD. The CVD burden is reduced by optimum diet through the replacement of unprocessed meat with low saturated fat, animal proteins, and plant proteins (76). The recommended Dietary Reference Intake (DRI) daily allowance in men aged 19–50 years is 38 g/day and women aged 19–50 years is 25 g/days, and for men aged >51 is 31 g/day and women aged >51 is 21 g/day. The recommendation for children aged 1–3 is 19 g/day and aged 4–8 is 25 g/day. For boys, aged 9–13, the DRI recommendations are 31 g/day and 38 g/days for aged 14–18. For girls aged 9–18, the DRI recommendations are 26 g/day (77). Individuals are also recommended to intake fruit and vegetables at least 5 servings per day, perform moderate activity for more than 150 min/week, do vigorous physical activity for at least 75 min/ week, reduce salt and sugar intake to prevent dyslipidemia (2).

## Limitations

The limitations of this systematic review with meta-analysis include some studies did not have enough predictor variables to adequately determine the degree of its prediction, However, attempts were made to include all other potential variables that occurred across the identified database. Another limitation of this study was that we did not identify the association between diet and dyslipidemia because we could not retrieve data from original articles. To overcome such problems, important discussions were made concerning the quantity and quality of daily intake. It might also lack continental representativeness since no information was found from northern regions of the African continent. But, the demographic characteristics of the population in the North African region were almost similar to the rest of the other regions.

## Conclusion

This review exposed that dyslipidemia is relatively high among study participants. The prevalence of dyslipidemia was the highest among the category of studies conducted on a group of communicable diseases and the general population. On the other hand, the lowest prevalence of dyslipidemia was reported among studies conducted on a group of infectious diseases. In addition, the highest prevalence of dyslipidemia was found among studies from East Africa and Southern Africa. The independent predictors of dyslipidemia were BMI >25.0 kg/m^2^, raised FBG, raised BP, and WC > 94 cm. In addition, sex, medication adherence, educational status, FBG, BP, BMI, WC, marital status, and sedentary life were strongly associated with dyslipidemia. There should also be a pressing public health measure to prevent, identify, and treat dyslipidemia with a special emphasis on obese, diabetic, and hypertensive patients. Future researchers were recommended to address the association between diet and dyslipidemia.

## Data Availability Statement

The original contributions presented in the study are included in the article/Supplementary Material, further inquiries can be directed to the corresponding author/s.

## Author Contributions

MO and GA were involved in the selection of study, data extraction, quality assessment, statistical analysis, results interpretation, and writing the initial and final drafts of the manuscript. GAA, BW, TT, NAw, NAy, YH, and BJ were involved in data extraction, quality assessment, statistical analysis, and writing drafts of the manuscript. All authors read and approved the final manuscript, contributed to the article, and approved the submitted version.

## Conflict of Interest

The authors declare that the research was conducted in the absence of any commercial or financial relationships that could be construed as a potential conflict of interest.

## Publisher's Note

All claims expressed in this article are solely those of the authors and do not necessarily represent those of their affiliated organizations, or those of the publisher, the editors and the reviewers. Any product that may be evaluated in this article, or claim that may be made by its manufacturer, is not guaranteed or endorsed by the publisher.

## Abbreviations

CI: confidence interval
HIV: human immune deficiency virus
HTN: hypertension
OR: odds ratio.
