# Supplementary material for: Determinants of Dyslipidemia in Africa: A Systematic Review and Meta-Analysis
Source: Front Cardiovasc Med. 2022 Feb 23;8:778891. doi: 10.3389/fcvm.2021.778891 (PMC8904727; doi:10.3389/fcvm.2021.778891)
Supplement: Supplementary file 1 [file Data_Sheet_1.DOCX]

| Concept 1 map | Dyslipidemia | 1.Key word | “dyslipid*”[tw] OR “dyslipidemia”[tw] OR “dyslipidaemia”[tw] OR “lipid profile”[tw]OR “Mets”[tw] OR “ metabolic syndrome ”[tw] OR “ Hypercholesterol* ”[tw] OR “ general population ”[tw] |
| --- | --- | --- | --- |
|  |  | 2.Mesh term | "dyslipidemias"[MeSH Terms] OR dyslipidemia[Text Word]"Hypercholesterolemia"[Mesh] |
| Concept 2 map | Prevalence | 3.Key word | “ Prevalence”[tw] OR “burden”[tw] |
|  |  | 4.Mesh term | ("Prevalence"[Mesh]) |
| Concept 3 map | Associated factors | 5.Key word | “Associated factors”[tw] OR “Risk factors”[tw] OR “Factors affecting”[tw] |
|  |  | 6.Mesh term | "Risk Factors"[Mesh] |
| Concept 4 map | Africa | 7.Key word | “Africa” [tw] OR “Sub-Saharan Africa”[tw] |
|  |  | 8.Mesh term | "africa"[MeSH Terms] OR Africa[Text Word] |

Determinants of dyslipidemia

("dyslipid*"[Text Word] OR "dyslipidemia"[Text Word] OR "dyslipidaemia"[Text Word] OR "lipid profile"[Text Word] OR "Mets"[Text Word] OR "metabolic syndrome"[Text Word] OR "hypercholesterol*"[Text Word] OR "general population"[Text Word] OR (("dyslipidemias"[MeSH Terms] OR "dyslipidemia"[Text Word]) AND "Hypercholesterolemia"[MeSH Terms])) AND ("Prevalence"[Text Word] OR "burden"[Text Word] OR "Prevalence"[MeSH Terms]) AND ("Associated factors"[Text Word] OR "risk factors"[Text Word] OR "Factors affecting"[Text Word] OR "risk factors"[MeSH Terms]) AND ("Africa"[Text Word] OR "Sub-Saharan Africa"[Text Word] OR ("Africa"[MeSH Terms] OR "Africa"[Text Word]))
